# COVID-19 Vaccination of Individuals with Down Syndrome—Data from the Trisomy 21 Research Society Survey on Safety, Efficacy, and Factors Associated with the Decision to Be Vaccinated

**DOI:** 10.3390/vaccines10040530

**Published:** 2022-03-29

**Authors:** Anke Hüls, Patrick T. Feany, Sophia Isabella Zisman, Alberto C. S. Costa, Mara Dierssen, Robert Balogh, Stefania Bargagna, Nicole T. Baumer, Ana Claudia Brandão, Angelo Carfi, Brian Allen Chicoine, Sujay Ghosh, Monica Lakhanpaul, Johannes Levin, Yona Lunsky, Coral Manso, Eitan Okun, Diego Real de Asua, Anne-Sophie Rebillat, Tilman R. Rohrer, Giuseppina Sgandurra, Diletta Valentini, Stephanie L. Sherman, Andre Strydom, on behalf of the Trisomy 21 Research Society COVID-19 Initiative

**Affiliations:** 1 Department of Epidemiology, Rollins School of Public Health, Emory University, Atlanta, GA 30322, USA; patrick.feany@emory.edu; 2 Gangarosa Department of Environmental Health, Rollins School of Public Health, Emory University, Atlanta, GA 30322, USA; 3 South London and Maudsley NHS Foundation Trust, London SE5 8AZ, UK; sophiaisabella.zisman@slam.nhs.uk (S.I.Z.); andre.strydom@kcl.ac.uk (A.S.); 4 Department of Psychiatry, Case Western Reserve University, Cleveland, OH 44106, USA; alberto.costa@case.edu; 5 Department of Macromolecular Science and Engineering, Case Western Reserve University, Cleveland, OH 44106, USA; 6 Centre for Genomic Regulation (CRG), The Barcelona Institute of Science and Technology, Universitat Pompeu Fabra, 08016 Barcelona, Spain; mara.dierssen@crg.eu; 7 Universitat Pompeu Fabra (UPF), 08016 Barcelona, Spain; 8 Centrode Investigación Biomédica en Red de Enfermedades Raras (CIBERER), 08016 Barcelona, Spain; 9 Faculty of Health Sciences, Ontario Tech University, Oshawa, ON L1G 0C5, Canada; robert.balogh@ontariotechu.ca; 10 Department of Developmental Neuroscience, IRCCS Fondazione Stella Maris, 56128 Calambrone, Italy; s.bargagna@fsm.unipi.it (S.B.); g.sgandurra@fsm.unipi.it (G.S.); 11 Department of Neurology, Division of Developmental Medicine, Department of Pediatrics, Boston Children’s Hospital, Harvard Medical School, Boston, MA 02115, USA; nicole.baumer@childrens.harvard.edu; 12 Hospital Israelita Albert Einstein, Sao Paulo 01000-000, SP, Brazil; ana.cbrandao@einstein.br; 13 Policlinico Universitario Agostino Gemelli IRCCS, 00100 Rome, Italy; angelo.carfi@policlinicogemelli.it; 14 Advocate Medical Group Adult Down Syndrome Center, Park Ridge, IL 60068, USA; brian.chicoine@aah.org; 15 Cytogenetics and Genomics Research Unit, Department of Zoology, University of Calcutta, Kolkata 700073, India; sgzoo@caluniv.ac.in; 16 Department of Population Policy and Practice, UCL Great Ormond Street Institute of Child Health, University College, London WC1N 1EH, UK; m.lakhanpaul@ucl.ac.uk; 17 Community Paediatrics, Whittington Health NHS, London N19 5NF, UK; 18 Department of Neurology, Ludwig-Maximilians-Universität München, 80539 Munich, Germany; johannes.levin@med.uni-muenchen.de; 19 German Center for Neurodegenerative Diseases, Site Munich, 53127 Munich, Germany; 20 Munich Cluster for Systems Neurology (SyNergy), 81377 Munich, Germany; 21 Azrieli Adult Neurodevelopmental Centre, CAMH, Temerty Faculty of Medicine, University of Toronto, Toronto, ON M5S 1A1, Canada; yona.lunsky@camh.ca; 22 Down España, 28001 Madrid, Spain; salud@sindromedown.net; 23 The Paul Feder Laboratory on Alzheimer’s Disease Research, Bar Ilan University, Ramat Gan 5290002, Israel; eitan.okun@biu.ac.il; 24 The Gonda Brain Research Center, Bar Ilan University, Ramat Gan 5290002, Israel; 25 The Mina and Everard Goodman Faculty of Life Sciences, Bar Ilan University, Ramat Gan 5290002, Israel; 26 Department of Internal Medicine and Instituto de Investigación Biomédica-La Princesa, Hospital Universitario de La Princesa, 28001 Madrid, Spain; diego.realdeasua@salud.madrid.org; 27 Institut Jérôme Lejeune, 75015 Paris, France; annesophie.rebillat@institutlejeune.org; 28 Division of Pediatric Endocrinology, Department of Pediatrics and Neonatology, Saarland University Medical Center, 66424 Homburg, Germany; tilman.rohrer@uks.eu; 29 Department of Clinical and Experimental Medicine, University of Pisa, 56126 Pisa, Italy; 30 Pediatric Unit, Pediatric Emergency Department, Bambino Gesù Children’s Hospital, IRCCS, 00165 Rome, Italy; diletta.valentini@opbg.net; 31 Department of Human Genetics, School of Medicine, Emory University, Atlanta, GA 30322, USA; ssherma@emory.edu; 32 Department of Forensic and Neurodevelopmental Sciences, Institute of Psychiatry, Psychology, and Neuroscience, King’s College London, London SE5 8AB, UK; 33 The London Down Syndrome (LonDownS) Consortium, London SE5 8AB, UK

**Keywords:** Trisomy 21, down syndrome, COVID-19, SARS-CoV-2, BNT162b2, mRNA-1273, ChAdOx1 nCoV-19, Ad26.COV2.S, vaccine hesitancy

## Abstract

Individuals with Down syndrome (DS) are among the groups with the highest risk for severe COVID-19. Better understanding of the efficacy and risks of COVID-19 vaccines for individuals with DS may help improve uptake of vaccination. The T21RS COVID-19 Initiative launched an international survey to obtain information on safety and efficacy of COVID-19 vaccines for individuals with DS. De-identified survey data collected between March and December 2021 were analyzed. Of 2172 individuals with DS, 1973 (91%) had received at least one vaccine dose (57% BNT162b2), 107 (5%) were unvaccinated by choice, and 92 (4%) were unvaccinated for other reasons. Most participants had either no side effects (54%) or mild ones such as pain at the injection site (29%), fatigue (12%), and fever (7%). Severe side effects occurred in <0.5% of participants. About 1% of the vaccinated individuals with DS contracted COVID-19 after vaccination, and all recovered. Individuals with DS who were unvaccinated by choice were more likely to be younger, previously recovered from COVID-19, and also unvaccinated against other recommended vaccines. COVID-19 vaccines have been shown to be safe for individuals with DS and effective in terms of resulting in minimal breakthrough infections and milder disease outcomes among fully vaccinated individuals with DS.

## 1. Introduction

Down syndrome (DS), the chromosomal disorder caused by the complete or partial trisomy of chromosome 21, is the most common genetic cause of intellectual disability worldwide, affecting around 1 in 732 live births [1]. DS is associated with immune dysregulation as well as several co-morbid health conditions that increase their risk for respiratory tract infections [2]. Individuals with DS are amongst the groups with the highest risk for hospitalization and mortality following infection with severe acute respiratory syndrome coronavirus 2 (SARS-CoV-2) [3,4,5,6]. Several studies have shown that hospitalized COVID-19 patients with DS are three to ten times more likely to die than those without DS [3,4,7]. Specific immune-dysregulation, accelerated aging, and co-morbid health conditions such as obesity, obstructive sleep apnea, diabetes, and congenital heart disease are likely contributory factors to this increase in risk [8]. In addition, there are several social factors that further increase these individuals’ risk of contracting COVID-19, such as persons with DS being more likely to live in residential care homes, where COVID-19 outbreaks have been well documented [9,10]. Therefore, implementing measures to protect this vulnerable patient group is of critical importance.

Vaccination programs are crucial for reducing morbidity and mortality from SARS-CoV-2 infection. As of 6 December 2021, the World Health Organization has issued emergency use listing for eight COVID-19 vaccinations, and over 7 billion vaccine doses have been administered worldwide [11]. Individuals with DS have been prioritized in several countries for urgent vaccination [12,13]. Vaccines have been deemed to be safe and effective against reducing mortality and hospitalization, and these benefits have been shown to outweigh the currently known side effects [14]. However, further study into COVID-19 vaccine response is needed in individuals with DS, as the immune dysregulation associated with the condition, specifically in response to viruses, combined with their complex comorbidity profiles may result in a different side effect profile and may also affect vaccine efficacy. For example, it has been shown that children with DS have a lower immune response to the influenza vaccination than children without DS [15,16] but a good immune response to a pneumococcal booster [16] and Hepatitis A vaccines [17].

Many individuals with DS have reduced capacity to make informed decisions about receiving the COVID-19 vaccination, which means that decisions may need to be made by caregivers or parents. A better understanding of the efficacy and risks of the COVID-19 vaccines may help provide individuals with DS and their caregivers factual information on which to base their decisions.

In order to obtain large-scale information on the safety and efficacy of the COVID-19 vaccines for individuals with DS and factors that influence the decision to receiving the vaccine, the Trisomy 21 Research Society (T21RS) launched an international online survey, with input from stakeholders including leading clinicians and DS organizations (details provided in the acknowledgements).

## 2. Methods

### 2.1. T21RS DS Survey

In March 2020, we developed an online survey to identify COVID-19 patients with DS to understand how they are affected by COVID-19 and if they are more vulnerable to severe COVID-19 than the general population. Details and results from our survey have been published previously [3,18,19].

Two equivalent surveys were developed, one to be completed by caregivers/family members of COVID-19-affected individuals with DS and one to be completed by clinicians. The survey collected information on: (1) basic demographics; (2) living situation during the pandemic; (3) pre-existing health conditions, vaccinations and medications; (4) SARS-CoV-2 testing; (5) COVID-19 signs and symptoms; (6) hospital and ICU admission; (7) complications associated with SARS-CoV-2 infection (clinician only); (8) medications and treatments used during COVID-19 illness (clinician only); and (9) status at last evaluation. The survey was implemented through the REDCap survey and database management system [20,21] and was hosted at Emory University.

In March 2021, we extended this initial survey by including questions related to the COVID-19 vaccination. In addition to the information above, our extended survey now includes questions related to vaccination status (e.g., vaccinated yes/no, how many doses of which vaccine, reasons for not being vaccinated), side effects after vaccination, and breakthrough infections.

The updated survey was translated into seven different languages and distributed in Europe, Asia, United States, Canada, and Latin America through clinical routes (e.g., Down syndrome medical interest group listservs and health service providers) and Down syndrome associations in the US, India, Spain, UK, France, Italy, Germany, Canada, Brazil, and Spanish-speaking Latin America, and DS registries (e.g., NIH DS-Connect^®^), as well as via the T21RS website. Each institution that planned to disseminate the survey within health services obtained IRB/ethics approval (Table S1). The study was performed according to the Declaration of Helsinki and national guidelines and regulations for data privacy, and all participants provided informed consent. All data were anonymized according to good clinical practice guidelines and data protection regulations. For this analysis, we included data from all participants that were entered between 1 March 2021 and 12 December 2021.

### 2.2. Statistical Analysis

Only individuals with information on age and gender were included in the analyses. We used descriptive statistics to show the study characteristics of the participants included in our analyses, stratified by vaccination status (vaccinated, unvaccinated by choice, unvaccinated for other reasons), and a description of side effects and their severity with additional information about the type of vaccine. To prevent study participants from being included twice in the analyses (reported by both caregiver and clinician), we excluded duplicated participants based on age, country/region, place of residence, ethnicity, trisomy type, and other specific demographics and comorbidities. In total, we identified 48 potential duplicates between the caregiver and clinician surveys, from which we only included the clinician information in the final analysis sample.

To identify factors associated with the decision not to be vaccinated among individuals with DS or their caregivers, we conducted adjusted logistic regression analyses with vaccination status as dependent variable (unvaccinated by choice versus vaccinated (reference category)). We investigated associations between vaccination status and age, gender, country of residence, level of intellectual disability, residential setting (living at home with family (reference group) versus other living conditions (living alone with support, small group home with support, residential care facility, other)), previous SARS-CoV-2 infection, vaccination status related to other optional vaccines (influenza, pneumococcal, human papillomavirus (HPV)), and various comorbidities as independent variables in individual regression analyses. We only included comorbidities that were present in at least 15% of our study samples to reduce the burden of multiple testing and the chance of false positives due to small numbers of cases. All associations were adjusted for age, gender, country, and residential setting unless one of these covariates was the independent variable of interest.

We performed all data analyses using R (version 4.1.1) [22].

## 3. Results

### 3.1. Description of Study Population

Of the 2172 records in our analysis sample entered between 1 March 2021 and 12 December 2021, 1973 individuals with DS were vaccinated (91%), 107 (5%) were unvaccinated by choice, and 92 (4%) were unvaccinated for other reasons (Table 1). Most data were reported by family members or caregivers (1792 (83%)) and the remaining data were entered by a clinician. Mean age (SD) was 28 (12) years (vaccinated: 28 (12) years, unvaccinated by choice: 21 (8) years, unvaccinated for other reasons: 22 (10) years) and 1173 (54%) were male. Most individuals with DS lived in the USA (836 (39%)), followed by Brazil (256 (12%)), Italy (246 (11%)), UK (224 (10%)), and Canada (134 (6%)). The majority of participants lived at home with their family (1825 (87%)), particularly among those who were unvaccinated (unvaccinated by choice: 102 (96%), unvaccinated for other reasons (76 (96%)). Overall, 280 of the 2172 participants (13%) had a previous SARS-CoV-2 infection. Of these, 259 participants (93%) had COVID-19 before being vaccinated. On average, the participants had 1.5 comorbidities (SD = 1.4), with thyroid disorders (1050 participants (53%)), obstructive sleep apnea (545 (29%)), and obesity (546 (29%)) being the most common comorbidities. Most participants were vaccinated against influenza (1494 (84%)). In addition, 742 (53%) were vaccinated against pneumococcal disease and 483 (35%) against HPV. These are optional but recommended vaccines in most countries.

**Figure 1:**
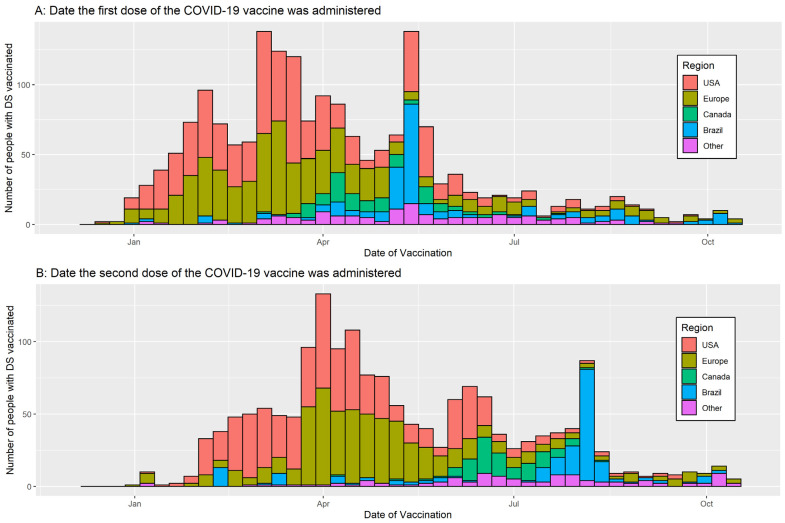
Date of vaccination stratified by first and second dose of the vaccine and country/region of residence.

Vaccination of individuals with DS started in December 2020 with the majority of participants from the USA and Europe being vaccinated in spring 2021 and those from Canada, Brazil, and India a few months later, in early summer 2021 (Figure 1).

### 3.2. Side Effects after the COVID-19 Vaccination

Overall, 1973 participants in the survey had received a first dose of a COVID-19 vaccine and 1723 had received a second dose. The majority received the Pfizer–BioNTech mRNA vaccine (BNT162b2; first dose: 1107 (56%), second dose: 1019 (59%)), followed by the Moderna mRNA vaccine (mRNA-1273; first dose: 318 (16%), second dose: 313 (18%)), Oxford-AstraZeneca adenovirus-vectored vaccine (ChAdOx1 nCoV-19; first dose: 355 (18%), second dose: 307 (18%)), and the Johnson & Johnson single-shot adenovirus-vectored vaccine (Ad26.COV2.S; first dose: 42 (2%)) (Table 2).

More than half of the vaccinated individuals with DS (first dose: 1048 (53%), second dose: 962 (56%)) did not experience any side effects after receiving a COVID-19 vaccine (Table 2). The most common reactions were pain at the site of injection (first dose: 578 (29% of those who received a first vaccine dose), second dose: 492 (29% of those who received a second vaccine dose)), fatigue (first dose: 217 (11%), second dose: 235 (14%)), fever (first dose: 183 (9%), second dose: 90 (5%)), redness at the site of injection (first dose: 151 (8%), second dose: 127 (7%)), headache (first dose: 148 (8%), second dose: 109 (6%)), muscle pain or body aches (first dose: 133 (7%), second dose: 92 (5%)), and chills (first dose: 72 (4%), second dose: 38 (2%)). Severe side effects were rare. Of the 1973 vaccinated individuals with DS, three (0.2%) developed myocarditis after the first dose of ChAdOx1 nCoV-19, and two individuals had blood clots (one individual after their second dose of BNT162b2 (0.1%) and one individual after their first dose of ChAdOx1 nCoV-19 (0.3%)).

For the mRNA vaccines (BNT162b2 and mRNA-1273), local reactions were similar between the two doses (e.g., BNT162b2 pain at injection site: first dose: 287 (26% of those who received a first BNT162b2 dose), second dose: 268 (26% of those who received a second BNT162b2 dose)), but systemic reactions, such as fatigue, fever, headache, and muscle pain or body aches, were more common after the second dose (Table 2). For ChAdOx1 nCoV-19, local as well as systemic reactions were more common after the first dose than after the second dose of the vaccine (e.g., pain at injection site: first dose: 149 (42%), second dose: 84 (27%); fever: first dose: 127 (36%), second dose: 14 (5%)).

Of the 1973 participants who received at least one dose of a COVID-19 vaccine, 183 (9%) had a SARS-CoV-2 infection prior to vaccination (Table 1). Side effects were similarly mild between participants with and without a prior SARS-CoV-2 infection (Table S2).

Most reactions did not require medical care (first dose: 766 (86%), second dose: 681 (93%)). Pain at the site of injection and fatigue, which were the most common side effects (Table 2), required medical care in less than 5% of the participants (Table 3). Of the 183 participants who had fever after the first dose of the vaccine, 17 (9%) saw a physician and 5 (3%) went to the emergency room or urgent care. After the second dose, three participants (3%) saw a physician after developing fever. Of the 1973 participants who received at least one dose of the vaccine, three participants were admitted to hospital or experienced other severe outcomes after vaccination. One participant developed blood clots after the second dose of BNT162b2. The patient was hospitalized for 23 days, but did not experience any resulting permanent morbid condition. The second participant had three seizures later than 3 months after the second dose of their COVID-19 vaccine (type of vaccine unknown). This individual had a history of seizures, and it could not be clinically confirmed whether the seizures were related to their vaccination. The third patient was admitted to hospital because of experiencing dizziness or fainting after the first dose of the vaccine (type of vaccine unknown).

### 3.3. Breakthrough Infections

The average time between receiving their latest dose of the vaccine and participation in the survey was 132 days (SD = 81). During this time period, a small number of vaccinated individuals with DS (21 people; 1%) contracted COVID-19 after vaccination and more than half of these infections occurred between the first and second dose of the vaccine (13 people), that is, before they were considered fully vaccinated (Table 1). Of these 13 participants, three had received their first dose of BNT162b2, seven had received their first dose of ChAdOx1 nCoV-19, and one had received their first dose of the Sinovac vaccine (others unknown; Table S3). Two of the thirteen participants with breakthrough infections after their first dose of a COVID-19 vaccine were hospitalized (one of them admitted to ICU), but none of them died and all had either fully recovered (eight participants) or were at least released from hospital at the time of the survey. Among the eight participants with breakthrough infections after being fully vaccinated, none were hospitalized and five had fully recovered at the time of the survey. One of the eight participants was asymptomatic and two had symptoms but remained non-hospitalized. Seven of the eight participants were vaccinated with BNT162b2 (other unknown; Table S3).

### 3.4. Factors Associated with Being Unvaccinated by Choice

**Figure 2:**
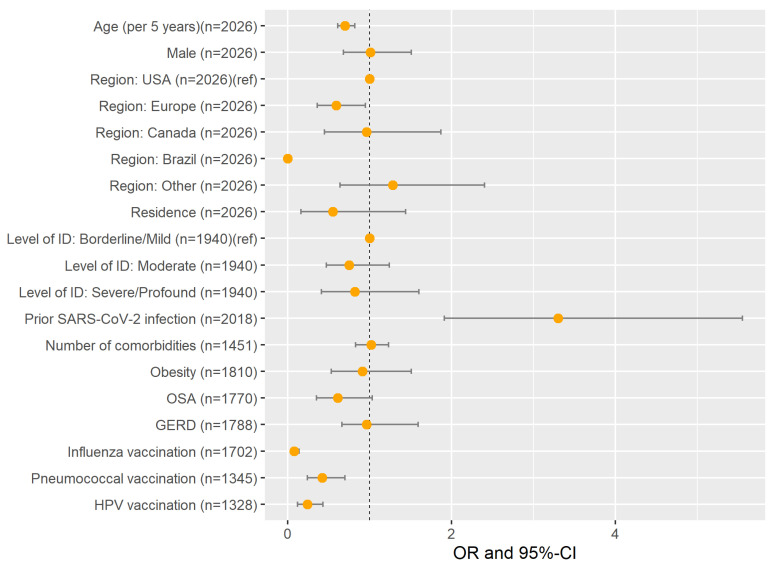
Factors associated with being unvaccinated by choice estimated with adjusted logistic regression models with vaccination status as dependent variable (unvaccinated by choice versus vaccinated [reference category]) (adjusted odds ratios (OR) and 95% confidence intervals (95% CI)). All associations were adjusted for age, gender, country, and residential setting unless one of these covariates was the independent variable of interest. Brazil had zero responses of being unvaccinated by choice. See Table 1 for details on how many participants were included from each European country. Other: Australia (n = 25), Colombia (n = 4), India (n = 104), Mexico (n = 6), Saudi Arabia (n = 5), and other countries with fewer than 4 participants. Residence: Living at home with family (reference group) versus other living conditions (see Table 1 for details on the other categories). For gender, the reference category was “female” (participants who identified neither as male nor female were excluded because of the low case number). For prior SARS-CoV-2 infection, comorbidities, and other vaccines, the references categories did not have those infection/comorbidities/vaccines. For factors with more than two categories, reference categories are included in the figure (ref). Comorbidities included in “number of comorbidities”: obesity, Alzheimer’s disease, thyroid disorder, epilepsy, blood cancer, other cancers, immuno-compromised, obstructive sleep apnea, hypertension, diabetes, cerebrovascular disease, coronary heart disease, chronic renal disease, chronic liver disease, chronic lung disease, celiac, gastroesophageal reflux, irritable bowel syndrome, and hepatitis B.

To understand factors associated with the decision to be vaccinated among individuals with DS, we conducted a logistic regression analysis comparing characteristics of vaccinated individuals with DS (N = 1973, reference category) against those who were unvaccinated by choice (N = 107), adjusting for confounding variables (see Figure 2 and Table S4 for details). Age, country of residence, previous COVID-19 disease, and being vaccinated against other optional vaccines (influenza, pneumococcal disease, HPV) were significantly associated with being unvaccinated by choice. Younger individuals with DS (odds ratios (OR) per 5 years increase [95% confidence interval] = 0.70 [0.61, 0.82]), those who had COVID-19 (3.30 [1.91, 5.55]), and those who had not received other optional but recommended vaccines were more likely to be unvaccinated by choice (influenza: 0.08 [0.05, 0.14], pneumococcal disease: 0.42 [0.24, 0.70], HPV: 0.24 [0.12, 0.43]). The proportion of individuals with DS who were unvaccinated by choice also differed by country. Individuals with DS living in the US (reference group) were more likely to be unvaccinated by choice than individuals from Europe (0.59 [0.36, 0.95]), but there was no significant difference in comparison to Canada (0.96 [0.45, 1.87]) or other countries (1.28 [0.64, 1.44]). Noteworthy, none of the 256 individuals with DS from Brazil were unvaccinated by choice. There were no significant differences in terms of comorbidities between vaccinated individuals with DS and those who were unvaccinated by choice and no significant difference in terms of residential setting or level of intellectual disability after adjusting for potential cofounding variables including age, gender, and country of residence.

## 4. Discussion

In this survey of 2172 individuals with DS, we showed that the COVID-19 vaccines are safe for this population and effective in terms of resulting in minimal breakthrough infections and milder disease outcomes among those fully vaccinated. Most participants had either no reaction or very mild reactions after vaccination. In total, 1% of the vaccinated individuals with DS contracted COVID-19 after vaccination and all of them either fully recovered or were at least released from hospital at the time of the survey. A total of 1973 (91%) participants in our survey had received at least one dose of the vaccine and 107 (5%) were unvaccinated by choice. Factors associated with being unvaccinated by choice included younger age, prior SARS-CoV-2 infection, country of residence, and being unvaccinated against other optional but recommended vaccines (pneumococcal disease, influenza, HPV), but not co-occurring health conditions.

Among the mild reactions to the COVID-19 vaccines, the most common side effect was pain at the injection site, which occurred among ~30% of participants. While there was no difference in this local reaction between the first and second dose of the mRNA vaccines (BNT162b2 and mRNA-1273), people with DS who received the viral vector vaccine ChAdOx1 nCoV-19 were more likely to experience pain at the injection site after the first dose (42%) than after the second dose (27%) of the vaccine. Local side effects reported for individuals with DS who received one of the mRNA vaccines are in line with data from a prospective observational study of 627,383 from the general population, showing that 29.2% and 34.3% of people who received the first and second dose of BNT162b2, respectively, experienced pain at the injection site [23]. The proportion of people with DS who experienced local pain as a side effect after receiving the first dose of ChAdOx1 nCoV-19 seems to be higher than reported in the general population (19% [23]). However, this finding needs to be interpreted with caution because of the relatively small number of participants in our survey who received ChAdOx1 nCoV-19 in comparison to mRNA vaccines. The proportion of people with DS who had mild-to-moderate systemic side effects (such as fatigue, fever, headache, muscle pain, or body aches and chills) was also similar to those observed in the general population [23]. However, the frequency of local and systemic side effects reported in this observational study of individuals with DS or the observational study of 627,383 from the general population [23] was lower than in the randomized clinical trials [24,25,26].

Severe side effects such as myocarditis and blood clots were rare, and all participants with DS experiencing any severe side effects fully recovered. Therefore, we conclude that the COVID-19 vaccines are safe for individuals with DS and the benefits definitely outweigh the risks, particularly given the high risk of hospitalization and death for individuals with DS infected with SARS-CoV-2 [3].

A small number of vaccinated individuals with DS (21 people; 1.1%) contracted COVID-19 after vaccination, and more than half of these infections occurred between the first and second dose of the vaccine (13 people), thus before they were considered fully vaccinated. None of these individuals died, and they all either fully recovered or were at least released from hospital at the time of the survey. This is in contrast to a recent report suggesting that vaccinated individuals with DS are still at high risk for hospitalization and death after being vaccinated [27]. However, their analyses are mainly based on infections that occurred between the first and second dose of the vaccine, thus before the participants were considered fully vaccinated, and the numbers of breakthrough infections were very low when stratifying by comorbidities, resulting in extremely wide confidence intervals [28].

A major threat to the impact of vaccination in preventing disease and death from COVID-19 is a lack of confidence in vaccines [29]. The reluctance of people to receive safe and recommended available vaccines, known as “vaccine hesitancy”, was already a growing concern before the COVID-19 pandemic [30]. Of note, the vaccination rate among people with DS who participated in our survey was 91%, which is remarkably high; though this proportion might be affected by sampling bias, assuming that people who were vaccinated were also more likely to participate in the survey. In our survey, individuals with DS were most likely to be unvaccinated by choice if they lived in the USA, followed by Canada. In contrast, none of the 248 individuals with DS from Brazil were unvaccinated by choice. This observation is in line with data from the general population, which show particularly high COVID-19 vaccine hesitancy among people from the USA, among other countries [31]. Other factors significantly associated with being unvaccinated by choice in our survey included younger age, prior SARS-CoV-2 infection, or being unvaccinated against other optional but recommended vaccines (pneumococcal disease, influenza, HPV).

Our survey showed that there was no significant difference in terms of number or severity of comorbidities when comparing individuals with DS who were vaccinated versus those who were unvaccinated by choice. Consequently, several of the individuals with DS who chose not to be vaccinated had severe comorbidities, which increases their risk of severe COVID-19 and death after infection with SARS-CoV-2 even more [3]. In contrast, a previous study of individuals with intellectual disabilities showed that having a health condition of concern in the context of COVID-19 increases the likelihood of receiving the vaccination [32]. However, our current study differs in terms of (1) only including individuals with DS, which is a known risk factor for severe COVID-19 itself in contrast to intellectual disabilities, and (2) including participants who have already been offered a vaccine in contrast to the previous study that focused on their intent to be vaccinated based on data from December 2020 to February 2021. Furthermore, our survey only included a small number of participants who were unvaccinated by choice (107 individuals with DS), which could have limited the statistical power of our analysis to detect differences in comorbidities.

Our observation that younger adults and children age 12–17 years with DS were more likely to be unvaccinated by choice is in line with data from the general population [29,33]. Reasons for this observation may include the fact that less time has passed since approval of the COVID-19 vaccines for children than for adults, which gave people less time to become familiar with the research on safety and efficacy of vaccines in this age group, and the general assumption that children and young adults do not become severely ill with COVID-19. However, our previous survey has shown that children with DS are at a much higher risk for severe COVID-19 than children from the general population [18], which demonstrates the importance of vaccinating children with DS against COVID-19.

A prior SARS-CoV-2 infection is another well-known factor influencing the decision to be vaccinated [34]. The US Centers for Disease Control and Prevention recommends COVID-19 vaccination for all eligible persons, including those who have been previously infected with SARS-CoV-2 [35]. While there is a wide range in antibody titers in response to infection with SARS-CoV-2, COVID-19 vaccines typically lead to a more consistent and higher-titer initial antibody response [35] and vaccination further enhances both humoral and cell-mediated immunity to variants in individuals with prior SARS-CoV-2 infection [36,37,38]. Given that the immune dysregulation associated with DS could lead to a shorter period of protection after vaccination or infection [2], it is particularly important for individuals with DS to be vaccinated against COVID-19, regardless of a prior infection with SARS-CoV-2.

Our finding that individuals with DS who were unvaccinated by choice were also more likely to not be vaccinated against other optional but recommended vaccines is in line with data from the general population [29] and with data from people with intellectual disabilities [32].

An important difference between vaccine hesitancy in the general population and vaccine hesitancy among people with intellectual disabilities (ID), including DS, is that the decision to be vaccinated is made by the individual with support of others, and in some cases, even with support, the person with DS cannot make the decision and it is made for them. A UK survey of adults with ID showed a high level of willingness to take a COVID-19 vaccine (87% of interviewed adults with ID) if it was offered to them [32]. Therefore, it is particularly important to educate caregivers and family members of individuals with DS about the severity of COVID-19 in unvaccinated individuals with DS and about the safety and efficacy of the COVID-19 vaccines in protecting individuals with DS against infection, severe COVID-19, and death.

Our study has several limitations. The survey includes participants from different countries, each country having its own profile with respect to the number of COVID-19 cases during the reporting period, the timing and prioritization within the rollout of the vaccine, the types of vaccines that are used, and a different presentation of SARS-CoV-2 variants. These differences could have affected the number of breakthrough infections that we observed across different countries as well as the time that has passed between being fully vaccinated and participation in the survey. In addition, as a sample of convenience, our study population might not be representative of the population of individuals with DS at large, especially in terms of vaccination rates (91% of the participants were vaccinated). Furthermore, as the average time between second dose and participation in our survey was 132 days, our current data cannot address the question of long-term protection against COVID-19 in individuals with DS. Another limitation of our study is that most information on side effects and their treatment was based on information provided by caregivers or family members of individuals with DS (83%). Therefore, information on rare side effects or their treatment should be interpreted with caution. In addition, because this was not a controlled clinical trial, we could not compare infection rates between vaccinated and unvaccinated people with DS. Therefore, our data on vaccine efficacy needs to be interpreted with caution. However, the low proportion of breakthrough infections we observed is in line with data from clinical trials and epidemiologic studies in the general population, and it supports the assumption that the COVID-19 vaccines are effective against infection with SARS-CoV-2 among people with DS. Unfortunately, we did not have information on repeated SARS-CoV-2 infections and were therefore not able to compare the frequency or severity of breakthrough infections among those who were vaccinated versus those who recovered from COVID-19.Our study did not include information on third doses of the vaccines (booster shots) or data from children younger than 12 years, and it was based on data from 1 March to 12 December 2021, a time window in which Alpha, Beta, and Delta were the dominant variants. We do not know how other variants (e.g., Omicron) would affect our conclusions. However, we have no reason to believe that the safety and efficacy of COVID-19 vaccines would be different between people with and without DS in light of new variants. Lastly, we had no information on socioeconomic factors or healthcare inequalities and income inequities, which are well known to be associated with the decision to be vaccinated [29]. However, it is probably safe to assume that these factors affect vaccine hesitancy among caregivers of individuals with DS in a similar way as they do in the general population.

## 5. Conclusions

In conclusion, our survey of 1973 vaccinated individuals with DS shows that the COVID-19 vaccines are safe and effective for individuals with DS, which is in line with data from the general population. As individuals with DS are at a very high risk for severe COVID-19 and death after infection with SARS-CoV-2, it is particularly important for them to be fully vaccinated against COVID-19 and to receive their booster as soon as eligible, regardless of their comorbidities or prior SARS-CoV-2 infection.

## Figures and Tables

**Table 1:** Study characteristics of the overall study population and subgroups stratified by vaccinated, unvaccinated by choice, and unvaccinated for other reasons.

	Overall	Vaccinated	Unvaccinated by Choice	Unvaccinated for Other Reasons ^1^
N	2172	1973	107	92
Age (mean (SD))	27.57 (12.09)	28.14 (12.14)	20.70 (7.89)	23.34 (11.78)
Gender (%)				
Female	996 (45.9)	911 (46.2)	50 (46.7)	35 (38.0)
Male	1173 (54.0)	1059 (53.7)	57 (53.3)	57 (62.0)
Other	3 (0.1)	3 (0.2)	0 (0.0)	0 (0.0)
Country (%)				
USA	836 (38.9)	758 (38.8)	58 (56.3)	20 (22.0)
Brazil	256 (11.9)	248 (12.7)	0 (0.0)	8 (8.8)
Italy	246 (11.5)	234 (12.0)	6 (5.8)	6 (6.6)
United Kingdom	224 (10.4)	211 (10.8)	7 (6.8)	6 (6.6)
Canada	134 (6.2)	122 (6.2)	10 (9.7)	2 (2.2)
France	127 (5.9)	124 (6.3)	2 (1.9)	1 (1.1)
India	104 (4.8)	71 (3.6)	0 (0.0)	33 (36.3)
Spain	62 (2.9)	60 (3.1)	1 (1.0)	1 (1.1)
Ireland (Republic)	29 (1.4)	23 (1.2)	3 (2.9)	3 (3.3)
Australia	25 (1.2)	21 (1.1)	3 (2.9)	1 (1.1)
Germany	18 (0.8)	12 (0.6)	6 (5.8)	0 (0.0)
Other ^2^	87 (4.1)	69 (3.5)	7 (6.8)	11 (12.0)
Level of intellectual disability (%)				
Borderline/Mild	542 (26.1)	495 (26.2)	27 (26.5)	20 (22.7)
Moderate	1254 (60.3)	1136 (60.2)	60 (58.8)	58 (65.9)
Severe/Profound	282 (13.6)	257 (13.6)	15 (14.7)	10 (11.4)
Type of Trisomy 21 (%)				
Full/standard	1815 (93.6)	1649 (93.7)	92 (95.8)	74 (89.2)
Mosaic	63 (3.2)	57 (3.2)	2 (2.1)	4 (4.8)
Translocation	56 (2.9)	50 (2.8)	2 (2.1)	4 (4.8)
Partial trisomy	5 (0.3)	4 (0.2)	0 (0.0)	1 (1.2)
Living situation before the COVID-19 outbreak (%)				
Living at home with family	1825 (86.5)	1642 (85.5	102 (96.2)	81 (96.4)
Living alone with support	63 (3.0)	61 (3.2)	2 (1.9)	0 (0.0)
Small group home with support	133 (6.3)	130 (6.8)	2 (1.9)	1 (1.2)
Residential care facility	45 (2.1)	45 (2.3)	0 (0.0)	0 (0.0)
Other	35 (1.7)	33 (1.7)	0 (0.0)	2 (2.4)
Living alone with no support (autonomously)	10 (0.5)	10 (0.5)	0 (0.0)	0 (0.0)
Survey completed by caregiver (%)	1792 (82.5)	1643 (83.3)	101 (94.4)	48 (52.2)
SARS-CoV-2 infection (%)				
Before vaccination	259 (11.9)	183 (9.3)	24 (22.4)	52 (56.5)
Between 1st and 2nd dose	13 (0.6)	13 (0.6)	0 (0.0)	0 (0.0)
After 2nd dose	8 (0.4)	8 (0.4)	0 (0.0)	0 (0.0)
Vaccinated for COVID-19 (%)	1973 (90.8)	1973 (100.0)	0 (0.0)	0 (0.0)
2nd dose of COVID vaccination (%)	1723 (92.6)	1723 (92.6)	NA	NA
Obesity (%)	546 (28.9)	490 (28.5)	21 (22.8)	35 (44.3)
Alzheimer disease/dementia (%)	94 (5.1)	86 (5.2)	0 (0.0)	8 (9.8)
Thyroid disorder (%)	1050 (52.7)	969 (53.4)	49 (51.6)	32 (38.1)
Seizures/epilepsy (%)	115 (6.2)	102 (6.0)	3 (3.4)	10 (12.2)
Blood cancer (%)	14 (0.8)	12 (0.7)	0 (0.0)	2 (2.5)
Other cancer (%)	6 (0.3)	4 (0.2)	0 (0.0)	2 (2.4)
Immuno-compromised (%)	53 (2.9)	40 (2.4)	1 (1.1)	12 (15.4)
Obstructive sleep apnea (%)	545 (29.4)	491 (29.2)	18 (20.5)	36 (45.0)
Hypertension (%)	47 (2.5)	33 (2.0)	2 (2.2)	12 (14.8)
Diabetes (%)	60 (3.2)	50 (3.0)	4 (4.5)	6 (7.3)
Cerebrovascular disease (%)	16 (0.9)	14 (0.8)	1 (1.1)	1 (1.2)
Coronary heart disease (%)	58 (3.1)	48 (2.9)	1 (1.1)	9 (11.4)
Chronic renal disease (%)	26 (1.4)	20 (1.2)	3 (3.3)	3 (3.8)
Chronic liver disease (%)	30 (1.6)	26 (1.6)	0 (0.0)	4 (4.9)
Chronic lung disease (%)	156 (8.4)	128 (7.6)	2 (2.2)	26 (32.9)
Celiac disease (%)	130 (7.0)	112 (6.6)	9 (10.1)	9 (11.4)
Gastroesophageal reflux (%)	260 (13.9)	232 (13.6)	11 (12.2)	17 (21.0)
Irritable bowel syndrome (%)	77 (4.2)	70 (4.2)	2 (2.3)	5 (6.2)
Hepatitis B infection (%)	5 (0.3)	5 (0.3)	0 (0.0)	0 (0.0)
Comorbid sum (mean (SD))	1.54 (1.39)	1.49 (1.33)	1.38 (1.30)	2.79 (2.11)
Other optional vaccines				
Influenza (%)	1494 (83.6)	1434 (86.6)	25 (34.7)	35 (58.3)
Pneumococcal (%)	742 (52.7)	701 (54.1)	22 (32.8)	19 (41.3)
HPV (%)	483 (34.7)	457 (36.0)	14 (19.4)	12 (24.0)

% were calculated after excluding missing information. ^1^ Other reasons include “not available yet” (n = 48), “medical reasons” (n = 24), “previous SARS-CoV-2 infection < 6 months ago” (n = 1), “planned (n = 4)”, “no reason given” (n = 11), “patent hard to treat” (n = 3), “death before vaccination” (n = 1). ^2^ Colombia (n = 4), Mexico (n = 6), Netherlands (n = 5), New Zealand (n = 5), Norway (n = 4), Portugal (n = 7), Saudi Arabia (n = 7), Sweden (n = 5), Switzerland (n = 6), and other countries with <4 participants.

**Table 2:** Reactions after receiving a COVID-19 vaccine stratified by vaccine and dose.

	Total		BNT162b2		mRNA-1273		ChAdOx1 nCoV-19		Ad26.COV2.S
	1st Dose	2nd Dose	1st Dose	2nd Dose	1st Dose	2nd Dose	1st Dose	2nd Dose	1st Dose
N	1973	1723	1107	1019	318	313	355	307	42
No reaction (%)	1048 (53.1)	962 (55.8)	727 (65.7)	622 (61.0)	161 (50.6)	127 (40.6)	105 (29.6)	180 (58.6)	22 (52.4)
Pain at injection site (%)	578 (29.3)	492 (28.6)	287 (25.9)	268 (26.3)	115 (36.2)	122 (39.0)	149 (42.0)	84 (27.4)	9 (21.4)
Fatigue (%)	217 (11.0)	235 (13.6)	99 (8.9)	135 (13.2)	47 (14.8)	77 (24.6)	54 (15.2)	22 (7.2)	12 (28.6)
Fever (%)	183 (9.3)	90 (5.2)	26 (2.3)	48 (4.7)	13 (4.1)	27 (8.6)	127 (35.8)	14 (4.6)	7 (16.7)
Redness at site (%)	151 (7.7)	127 (7.4)	58 (5.2)	55 (5.4)	39 (12.3)	36 (11.5)	46 (13.0)	29 (9.4)	1 (2.4)
Headache (%)	148 (7.5)	109 (6.3)	37 (3.3)	55 (5.4)	20 (6.3)	33 (10.5)	86 (24.2)	20 (6.5)	2 (4.8)
Muscle pain or body aches (%)	133 (6.7)	92 (5.3)	29 (2.6)	47 (4.6)	23 (7.2)	32 (10.2)	71 (20.0)	13 (4.2)	7 (16.7)
Chills (%)	72 (3.6)	38 (2.2)	7 (0.6)	12 (1.2)	5 (1.6)	16 (5.1)	56 (15.8)	10 (3.3)	3 (7.1)
Nausea, vomiting or diarrhea (%)	49 (2.5)	20 (1.2)	7 (0.6)	9 (0.9)	6 (1.9)	8 (2.6)	35 (9.9)	3 (1.0)	1 (2.4)
Dizziness or fainting (%)	37 (1.9)	14 (0.8)	7 (0.6)	7 (0.7)	2 (0.6)	4 (1.3)	25 (7.0)	2 (0.7)	1 (2.4)
Skin reaction (%)	22 (1.1)	8 (0.5)	8 (0.7)	3 (0.3)	3 (0.9)	2 (0.6)	10 (2.8)	2 (0.7)	0 (0)
Shortness of breath (%)	14 (0.7)	4 (0.2)	2 (0.2)	0 (0)	1 (0.3)	1 (0.3)	8 (2.3)	2 (0.7)	1 (2.4)
Hypotension (%)	14 (0.7)	3 (0.2)	4 (0.4)	1 (0.1)	0 (0)	1 (0.3)	9 (2.5)	1 (0.3)	0 (0)
Tachycardia (%)	12 (0.6)	3 (0.2)	1 (0.1)	0 (0)	0 (0)	1 (0.3)	10 (2.8)	1 (0.3)	1 (2.4)
Adenopathies (%)	7 (0.4)	2 (0.1)	2 (0.2)	1 (0.1)	1 (0.3)	1 (0.3)	3 (0.8)	0 (0)	0 (0)
Allergic reaction (%)	6 (0.3)	1 (0.1)	1 (0.1)	0 (0)	1 (0.3)	0 (0)	2 (0.6)	0 (0)	1 (2.4)
Wheezing (%)	4 (0.2)	1 (0.1)	0 (0)	0 (0)	2 (0.6)	0 (0)	2 (0.6)	1 (0.3)	0 (0)
Myocarditis (%)	3 (0.2)	0 (0)	0 (0)	0 (0)	0 (0)	0 (0)	3 (0.8)	0 (0)	0 (0)
Swelling of face, lips or throat (%)	4 (0.2)	1 (0.1)	1 (0.1)	0 (0)	1 (0.3)	0 (0)	1 (0.3)	0 (0)	0 (0)
Worsening/new diabetic symptoms (%)	2 (0.1)	2 (0.1)	2 (0.2)	1 (0.1)	0 (0)	1 (0.3)	0 (0)	0 (0)	0 (0)
Bell’s Palsy (%)	1 (0.1)	0 (0)	0 (0)	0 (0)	1 (0.3)	0 (0)	0 (0)	0 (0)	0 (0)
Blood clot (%)	1 (0.1)	1 (0.1)	0 (0)	1 (0.1)	0 (0)	0 (0)	1 (0.3)	0 (0)	0 (0)
Blood clot in brain (%)	0 (0)	0 (0)	0 (0)	0 (0)	0 (0)	0 (0)	0 (0)	0 (0)	0 (0)
Other (%)	25 (1.3)	27 (1.6)	12 (1.1)	14 (1.4)	6 (1.9)	7 (2.2)	7 (2.0)	5 (1.6	0 (0)
Do not Know (%)	16 (0.8)	19 (1.1)	7 (0.6)	8 (0.8)	3 (0.9)	5 (1.6)	3 (0.8)	6 (2.0)	1 (2.4)

Numbers presented as n (%) of total doses administered stratified by 1st and 2nd dose of the vaccine; % were calculated after excluding missing information.

**Table 3:** Severity of reactions. Number (%) of people who received medical care after receiving a COVID-19 vaccine stratified by type of side effects that required medical care.

	Total		No Medical Care Needed		Doctor Visit		ER/Urgent Care		Admitted to Hospital or Other Severe Outcomes ^1^	
Dose of vaccine	1st	2nd	1st	2nd	1st	2nd	1st	2nd	1st	2nd
n	900	734	766 (86.2)	681 (92.8)	38 (4.2)	14 (1.9)	8 (0.8)	3 (0.4)	1 (0.1)	3 (0.4)
Pain at site	578	493	548 (94.8)	482 (97.8)	22 (3.8)	7 (1.4)	6 (1.0)	1 (0.2)	0 (0)	0 (0)
Fatigue	217	236	205 (94.5)	229 (97.0)	7 (3.2)	2 (0.8)	2 (0.9)	0 (0)	0 (0)	1 (0.4) ^2^
Fever	183	90	162 (88.5)	83 (92.2)	17 (9.3)	3 (3.3)	5 (2.7)	1 (1.1)	0 (0)	1 (1.1) ^2^
Redness at site	151	128	133 (88.1)	121 (94.5)	14 (9.3)	6 (4.7)	5 (3.3)	0 (0)	0 (0)	0 (0)
Headache	148	109	130 (87.8)	102 (93.6)	14 (9.5)	4 (3.7)	6 (4.1)	1 (0.9)	0 (0)	1 (0.9) ^2^
Muscle pain or body aches	133	92	120 (90.2)	87 (94.6)	8 (6.0)	3 (3.3)	5 (3.8)	0 (0)	0 (0)	1 (1.1) ^2^
Chills	72	38	61 (84.7)	35 (92.1)	8 (11.1)	2 (5.3)	4 (5.6)	0 (0)	0 (0)	0 (0)
Nausea, vomiting or diarrhea	49	20	37 (75.5)	19 (95.0)	8 (16.3)	1 (5.0)	3 (6.1)	0 (0)	0 (0)	0 (0)
Dizziness or fainting	37	14	27 (73.0)	12 (85.7)	7 (18.9)	2 (14.3)	4 (10.8)	0 (0)	1 (2.7) ^3^	0 (0)
Skin reaction	22	8	15 (68.2)	4 (50.0)	6 (27.3)	3 (37.5)	2 (9.1)	1 (12.5)	0 (0)	0 (0)
Shortness of breath	14	4	7 (50.0)	3 (75.0)	5 (35.7)	0 (0)	2 (14.3)	0 (0)	0 (0)	0 (0)
Hypotension	14	3	7 (50.0)	1 (33.3)	4 (28.6)	2 (66.7)	4 (28.6)	0 (0)	0 (0)	0 (0)
Tachycardia	12	3	8 (66.7)	2 (66.7)	3 (25.0)	0 (0)	2 (16.7)	0 (0)	0 (0)	1 (33.3) ^2^
Adenopathies	7	2	6 (85.7)	2 (100)	1 (14.3)	0 (0)	0 (0)	0 (0)	0 (0)	0 (0)
Allergic reaction	6	1	3 (50.0)	1 (100)	3 (50.0)	0 (0)	0 (0)	0 (0)	0 (0)	0 (0)
Wheezing	4	1	2 (50.0)	0 (0)	1 (25.0)	1 (100)	1 (25.0)	0 (0)	0 (0)	0 (0)
Myocarditis	3	0	2 (66.7)	0 (0)	0 (0)	0 (0)	1 (33.3)	0 (0)	0 (0)	0 (0)
Swelling of face, lips or throat	4	1	1 (25.0)	0 (0)	1 (25.0)	1 (100)	2 (50.0)	0 (0)	0 (0)	0 (0)
Worsening or new diabetic symptoms	2	2	2 (100)	0 (0)	0 (0)	2 (100)	0 (0)	0 (0)	0 (0)	0 (0)
Bell’s Palsy	1	0	0 (0)	0 (0)	0 (0)	0 (0)	0 (0)	0 (0)	0 (0)	0 (0)
Blood clot	1	1	0 (0)	0 (0)	1 (100)	0 (0)	0 (0)	0 (0)	0 (0)	1 (100) ^4^
Blood clot in brain	0	0	0 (0)	0 (0)	0 (0)	0 (0)	0 (0)	0 (0)	0 (0)	0 (0)
Other	25	27	18 (72.0)	21 (77.8)	5 (20.0)	4 (14.8)	0 (0)	1 (3.7) ^5^	0 (0)	1 (3.7) ^5^

Numbers presented as n (%) of total doses administered stratified by 1st and 2nd dose of the vaccine; % were calculated after excluding missing information. Participants were asked to select all options that apply. Therefore, the row-wise percentages do not sum up to 100%. ^1^ Combined levels Admitted to Hospital, Longer hospital stay, ICU/life threatening reaction, Permanent damage, Death. ^2^ No reaction to first shot, 8 days in hospital, recovered. ^3^ Only reaction to vaccination, recovered and received 2nd dose a month later, reported fatigue after second vaccination. ^4^ 23 days hospitalized for blood clot, only reaction. No reaction to first dose. No permanent damage. ^5^ had 3 seizures > 3 months after 2nd dose of vaccine. Had a history of seizures. No manufacturer is listed.

## Data Availability

Written requests for the use of the anonymized data collected through the T21RS survey will be reviewed for approval by the T21RS COVID-19 Initiative.

## Supplementary Materials

The following supporting information can be downloaded at: https://www.mdpi.com/article/10.3390/vaccines10040530/s1, Complete list of the Trisomy 21 Research Society COVID-19 Initiative group members; Table S1: Each institution that planned to disseminate the survey obtained IRB/ethics approval; Table S2: Side effects after the COVID-19 vaccination stratified by vaccine type (viral vector: ChAdOx1 nCoV-19, Ad26.COV2.S; mRNA: BNT162b2, mRNA-1273), dose and prior SARS-CoV-2 infection; Table S3: Description of SARS-CoV-2 breakthrough infections observed among vaccinated people with Down syndrome; Table S4: Factors associated with being unvaccinated by choice.

## References

[1] Mai C.T., Isenburg J.L., Canfield M.A., Meyer R.E., Correa A., Alverson C.J., Lupo P.J., Riehle-Colarusso T., Cho S.J., Aggarwal D. (2019). National population-based estimates for major birth defects, 2010–2014. Birth Defects Res..

[2] Illouz T., Biragyn A., Iulita M.F., Flores-Aguilar L., Dierssen M., De Toma I., Antonarakis S.E., Yu E., Herault Y., Potier M.C. (2021). Immune Dysregulation and the Increased Risk of Complications and Mortality Following Respiratory Tract Infections in Adults With Down Syndrome. Front. Immunol..

[3] Hüls A., Costa A.C.S., Dierssen M., Baksh R.A., Bargagna S., Baumer N.T., Brandão A.C., Carfi A., Carmona-Iragui M., Chicoine B.A. (2021). Medical vulnerability of individuals with Down syndrome to severe COVID-19-data from the Trisomy 21 Research Society and the UK ISARIC4C survey. eClinicalMedicine.

[4] Clift A.K., Coupland C.A.C., Keogh R.H., Hemingway H., Hippisley-Cox J. (2021). COVID-19 Mortality Risk in Down Syndrome: Results from a Cohort Study of 8 Million Adults. Ann. Intern. Med..

[5] Malle L., Gao C., Hur C., Truong H.Q., Bouvier N.M., Percha B., Kong X.F., Bogunovic D. (2021). Individuals with Down syndrome hospitalized with COVID-19 have more severe disease. Genet. Med..

[6] Williamson E.J., McDonald H.I., Bhaskaran K., Walker A.J., Bacon S., Davy S., Schultze A., Tomlinson L., Bates C., Ramsay M. (2021). Risks of COVID-19 hospital admission and death for people with learning disability: Population based cohort study using the OpenSAFELY platform. BMJ.

[7] Lunsky Y., Durbin A., Balogh R., Lin E., Palma L., Plumptre L. (2021). COVID-19 positivity rates, hospitalizations and mortality of adults with and without intellectual and developmental disabilities in Ontario, Canada. Disabil. Health J..

[8] Illouz T., Biragyn A., Frenkel-Morgenstern M., Weissberg O., Gorohovski A., Merzon E., Green I., Iulita F., Flores-Aguilar L., Dierssen M. (2021). Specific Susceptibility to COVID-19 in Adults with Down Syndrome. NeuroMolecular Med..

[9] Stancliffe R.J., Charlie Lakin K., Larson S.A., Engler J., Taub S., Fortune J., Bershadsky J. (2012). Demographic characteristics, health conditions, and residential service use in adults with down syndrome in 25 U.S. states. Intellect. Dev. Disabil..

[10] McMichael T.M., Currie D.W., Clark S., Pogosjans S., Kay M., Schwartz N.G., Lewis J., Baer A., Kawakami V., Lukoff M.D. (2020). Epidemiology of COVID-19 in a long-term care facility in King County, Washington. N. Engl. J. Med..

[11] WHO WHO Coronavirus (COVID-19) Dashboard. https://covid19.who.int/.

[12] CDC People with Certain Medical Conditions. https://www.cdc.gov/coronavirus/2019-ncov/need-extra-precautions/people-with-medical-conditions.html.

[13] NHS Who Is at High Risk from Coronavirus (COVID-19). https://www.nhs.uk/conditions/coronavirus-covid-19/people-at-higher-risk/who-is-at-high-risk-from-coronavirus/.

[14] CDC Science Brief: COVID-19 Vaccines and Vaccination. https://www.cdc.gov/coronavirus/2019-ncov/science/science-briefs/fully-vaccinated-people.html.

[15] Kusters M.A., Bok V.L.A., Bolz W.E.A., Huijskens E.G.W., Peeters M.F., De Vries E. (2012). Influenza A/H1N1 vaccination response is inadequate in Down syndrome children when the latest cut-off values are used. Pediatr. Infect. Dis. J..

[16] Valentini D., Marcellini V., Bianchi S., Villani A., Facchini M., Donatelli I., Castrucci M.R., Marasco E., Farroni C., Carsetti R. (2015). Generation of switched memory B cells in response to vaccination in Down syndrome children and their siblings. Vaccine.

[17] Ferreira C.T., Leite J.C., Taniguchi A., Vieira S.M., Pereira-Lima J., da Silveira T.R. (2004). Immunogenicity and safety of an inactivated hepatitis a vaccine in children with down syndrome. J. Pediatr. Gastroenterol. Nutr..

[18] Emes D., Hüls A., Baumer N., Dierssen M., Puri S., Russell L., Sherman S.L., Strydom A., Bargagna S., Brandão A.C. (2021). COVID-19 in Children with Down Syndrome: Data from the Trisomy 21 Research Society Survey. J. Clin. Med..

[19] Real de Asua D., Mayer M.A., Ortega M.D.C., Borrel J.M., de Bermejo T.J., González-Lamuño D., Manso C., Moldenhauer F., Carmona-Iragui M., Hüls A. (2021). Comparison of COVID-19 and Non-COVID-19 Pneumonia in Down Syndrome. J. Clin. Med..

[20] Harris P.A., Taylor R., Thielke R., Payne J., Gonzalez N., Conde J.G. (2009). Research electronic data capture (REDCap)-A metadata-driven methodology and workflow process for providing translational research informatics support. J. Biomed. Inform..

[21] Harris P.A., Taylor R., Minor B.L., Elliott V., Fernandez M., O’Neal L., McLeod L., Delacqua G., Delacqua F., Kirby J. (2019). The REDCap consortium: Building an international community of software platform partners. J. Biomed. Inform..

[22] R Core Team (2020). R: A Language and Environment for Statistical Computing.

[23] Menni C., Klaser K., May A., Polidori L., Capdevila J., Louca P., Sudre C.H., Nguyen L.H., Drew D.A., Merino J. (2021). Vaccine side-effects and SARS-CoV-2 infection after vaccination in users of the COVID Symptom Study app in the UK: A prospective observational study. Lancet Infect. Dis..

[24] Polack F.P., Thomas S.J., Kitchin N., Absalon J., Gurtman A., Lockhart S., Perez J.L., Pérez Marc G., Moreira E.D., Zerbini C. (2020). Safety and Efficacy of the BNT162b2 mRNA COVID-19 Vaccine. N. Engl. J. Med..

[25] Baden L.R., El Sahly H.M., Essink B., Kotloff K., Frey S., Novak R., Diemert D., Spector S.A., Rouphael N., Creech C.B. (2021). Efficacy and Safety of the mRNA-1273 SARS-CoV-2 Vaccine. N. Engl. J. Med..

[26] Sadoff J., Gray G., Vandebosch A., Cárdenas V., Shukarev G., Grinsztejn B., Goepfert P.A., Truyers C., Fennema H., Spiessens B. (2021). Safety and Efficacy of Single-Dose Ad26.COV2.S Vaccine against COVID-19. N. Engl. J. Med..

[27] Hippisley-Cox J., Coupland C.A.C., Mehta N., Keogh R.H., Diaz-Ordaz K., Khunti K., Lyons R.A., Kee F., Sheikh A., Rahman S. (2021). Risk prediction of COVID-19 related death and hospital admission in adults after COVID-19 vaccination: National prospective cohort study. BMJ.

[28] Strydom A., Costa A., Hüls A., Sherman S., Lunsky Y. Rapid Response: Re: Risk prediction of COVID-19 related death and hospital admission in adults after COVID-19 vaccination: National prospective cohort study. *BMJ*
**2021**, *374*, n2244. https://www.bmj.com/content/374/bmj.n2244/rr-2.

[29] Razai M.S., Chaudhry U.A.R., Doerholt K., Bauld L., Majeed A. (2021). COVID-19 vaccination hesitancy. BMJ.

[30] MacDonald N.E. (2015). SAGE Working Group on Vaccine Hesitancy Vaccine hesitancy: Definition, scope and determinants. Vaccine.

[31] Sallam M. (2021). COVID-19 vaccine hesitancy worldwide: A concise systematic review of vaccine acceptance rates. Vaccines.

[32] Hatton C., Bailey T., Bradshaw J., Caton S., Flynn S., Gillooly A., Jahoda A., Maguire R., Marriott A., Mulhall P. (2021). The willingness of UK adults with intellectual disabilities to take COVID-19 vaccines. J. Intellect. Disabil. Res..

[33] Willis D.E., Andersen J.A., Bryant-Moore K., Selig J.P., Long C.R., Felix H.C., Curran G.M., McElfish P.A. (2021). COVID-19 vaccine hesitancy: Race/ethnicity, trust, and fear. Clin. Transl. Sci..

[34] Gerussi V., Peghin M., Palese A., Bressan V., Visintini E., Bontempo G., Graziano E., De Martino M., Isola M., Tascini C. (2021). Vaccine hesitancy among italian patients recovered from COVID-19 infection towards influenza and SARS-CoV-2 vaccination. Vaccines.

[35] CDC Science Brief: SARS-CoV-2 Infection-Induced and Vaccine-Induced Immunity. https://www.cdc.gov/coronavirus/2019-ncov/science/science-briefs/vaccine-induced-immunity.html.

[36] Urbanowicz R.A., Tsoleridis T., Jackson H.J., Cusin L., Duncan J.D., Chappell J.G., Tarr A.W., Nightingale J., Norrish A.R., Ikram A. (2021). Two doses of the SARS-CoV-2 BNT162b2 vaccine enhance antibody responses to variants in individuals with prior SARS-CoV-2 infection. Sci. Transl. Med..

[37] Reynolds C.J., Pade C., Gibbons J.M., Butler D.K., Otter A.D., Menacho K., Fontana M., Smit A., Sackville-West J.E., Cutino-Moguel T. (2021). Prior SARS-CoV-2 infection rescues B and T cell responses to variants after first vaccine dose. Science.

[38] Lozano-Ojalvo D., Camara C., Lopez-Granados E., Nozal P., Del Pino-Molina L., Bravo-Gallego L.Y., Paz-Artal E., Pion M., Correa-Rocha R., Ortiz A. (2021). Differential effects of the second SARS-CoV-2 mRNA vaccine dose on T cell immunity in naive and COVID-19 recovered individuals. Cell Rep..

