# Supplementary material for: COVID-19 Vaccination of Individuals with Down Syndrome—Data from the Trisomy 21 Research Society Survey on Safety, Efficacy, and Factors Associated with the Decision to Be Vaccinated
Source: Vaccines (Basel). 2022 Mar 29;10(4):530. doi: 10.3390/vaccines10040530 (PMC9030605; doi:10.3390/vaccines10040530)
Supplement: Supplementary file 1 [file vaccines-10-00530-s001.zip › T21RS Group Authors Affiliations.pdf]

First Initials	Surname	Affiliation
TdJ	Bermejo	Instituto Hispalense de Psiquiatría, Spain
P	Borrell	DOWN ESPAÑA, Madrid, Spain
L	Cretu	Institut Jérôme Lejeune, Paris, France
R	de la Torre	Hospital del Mar Medical Research Institute, Spain
J	Florez	Fundacion Iberoamericana Down 21, Spain
J	Fortea	Sant Pau Memory Unit, Department of Neurology, Hospital de la Santa Creu i Sant Pau, Biomedical Research Institute Sant Pau, Universitat Autònoma de Barcelona, Barcelona, Spain; Barcelona Down Medical Center, Fundació Catalana de Síndrome de Down, Barcelona, Spain
P	Ghosh	Department of Zoology. Bijoy Krishna Girls' College. Howrah, West Bengal, India
D	González-Lamuño	Hospital Valdecilla, Santander, Cantabria, Spain
A	Hiance-Delahaye	Institut Jérôme Lejeune, Paris, France
C	Laffon	Institut Jérôme Lejeune, Paris, France
A	Matia	DOWN ESPANA, Barcelona, Spain
C	Mircher	Institut Jérôme Lejeune, Paris, France
F	Moldenhauer	Hospital La Princesa, Madrid, Spain
M	Mulqueen	Advantage Care Health Centers, Brookville, NY, USA
NJ	Nowalk	Children’s Hospital of Pittsburgh, Pittsburgh, PA, USA
E	Prioux	Institut Jérôme Lejeune, Paris, France
F	Sanz	Research Programme on Biomedical Informatics, Hospital del Mar and DCEXS Universitat Pompeu Fabra, Barcelona, Spain
J	Toulas	Institut Jérôme Lejeune, Paris, France
