# Supplementary material for: COVID-19 Vaccination of Individuals with Down Syndrome—Data from the Trisomy 21 Research Society Survey on Safety, Efficacy, and Factors Associated with the Decision to Be Vaccinated
Source: Vaccines (Basel). 2022 Mar 29;10(4):530. doi: 10.3390/vaccines10040530 (PMC9030605; doi:10.3390/vaccines10040530)
Supplement: Supplementary file 1 [file vaccines-10-00530-s001.zip › vaccines-1630190_supplement_final.pdf]

Supplementary Material

Table S1. Each institution that planned to disseminate the survey obtained IRB/ethics approval.

**Spain**	The study was approved by the Hospital del Mar ethics committee (CEIC Parc de Salut Mar, CEim 2020/9197)
**United Kingdom, Canada**	The T21RS survey was approved by the Health research agency (HRA) 20/HRA/2452.
**Brazil**	This study was approved by the Brazilian Federal Ethics Committee (CONEP, CAAE: 30847520.8.0000.0071)
**Emory University, U.S.A.**	This study was deemed exempt from human subjects research under 45 CFR 46.104(d)(2i) (IRB ID: STUDY00000386)
**Italy**	The study was approved by the Bambino Gesù children's Hospital Ethics Committee (2091_OPBG_2020)
**Israel**	The Ethics Committee constituted by Bar Ilan University determined this study to have Exempt Status.
**Advocate Health Care Institutional Review Board**	Determined to have Exempt Status IRB# 20-151ET
**France**	This study was approved by CPP Sud Mediterranée IV dated 29/04/2020 (ID RCB 2020-A00940-39)
**Ludwig-Maximilians-Universität (LMU), Munich, Germany**	Determined to have Exempt Status (IRB ID: 20-573 KB)
**India**	The study was approved by the Ethics Committee constituted by the University of Calcutta (CU/BIOETHICS/HUMAN/2306/3044/2020)

Table S2. Side effects after the COVID-19 vaccination stratified by vaccine type (viral vector: ChAdOx1 nCoV-19, Ad26.COV2.S; mRNA: BNT162b2, mRNA-1273), dose and prior SARS-CoV-2 infection.

	1st dose				2nd dose			
	Viral vector vaccine		mRNA vaccine		Viral vector vaccine		mRNA vaccine	
	No prior SARS-CoV-2 infection	Prior SARS-CoV-2 infection	No prior SARS-CoV-2 infection	Prior SARS-CoV-2 infection	No prior SARS-CoV-2 infection	Prior SARS-CoV-2 infection	No prior SARS-CoV-2 infection	Prior SARS-CoV-2 infection
n	347	59	1330	95	287	40	1280	68
No reaction (%)	113 (32.6)	20 (33.9)	829 (62.3)	59 (62.1)	170 (59.2)	19 (47.5)	705 (55.1)	42 (61.8)
Pain at site (%)	137 (39.5)	23 (39.0)	377 (28.3)	25 (26.3)	72 (25.1)	16 (40.0)	369 (28.8)	18 (26.5)
Fatigue (%)	64 (18.4)	2 (3.4)	134 (10.1)	12 (12.6)	23 (8.0)	0 (0.0)	202 (15.8)	10 (14.7)
Fever (%)	123 (35.4)	12 (20.3)	31 (2.3)	8 (8.4)	11 (3.8)	3 (7.5)	69 (5.4)	6 (8.8)
Redness at site (%)	34 (9.8)	13 (22.0)	85 (6.4)	12 (12.6)	19 (6.6)	10 (25.0)	85 (6.6)	7 (10.3)
Headache (%)	80 (23.1)	8 (13.6)	49 (3.7)	8 (8.4)	18 (6.3)	2 (5.0)	84 (6.6)	4 (5.9)
Muscle pain or body aches (%)	75 (21.6)	4 (6.8)	43 (3.2)	9 (9.5)	11 (3.8)	2 (5.0)	74 (5.8)	5 (7.4)
Chills (%)	55 (15.9)	4 (6.8)	9 (0.7)	3 (3.2)	10 (3.5)	0 (0.0)	26 (2.0)	2 (2.9)
Nausea, vomiting or diarrhea (%)	34 (9.8)	2 (3.4)	12 (0.9)	1 (1.1)	3 (1.0)	0 (0.0)	17 (1.3)	0 (0.0)
Dizziness or fainting (%)	19 (5.5)	7 (11.9)	9 (0.7)	0 (0.0)	2 (0.7)	0 (0.0)	11 (0.9)	0 (0.0)
Skin reaction (%)	4 (1.2)	6 (10.2)	11 (0.8)	0 (0.0)	2 (0.7)	0 (0.0)	5 (0.4)	0 (0.0)
Shortness of breath (%)	6 (1.7)	3 (5.1)	1 (0.1)	2 (2.1)	0 (0.0)	2 (5.0)	1 (0.1)	0 (0.0)
Hypotension (%)	7 (2.0)	2 (3.4)	3 (0.2)	1 (1.1)	0 (0.0)	1 (2.5)	2 (0.2)	0 (0.0)
Tachycardia (%)	9 (2.6)	2 (3.4)	1 (0.1)	0 (0.0)	1 (0.3)	0 (0.0)	1 (0.1)	0 (0.0)
Adenopathies (%)	2 (0.6)	1 (1.7)	3 (0.2)	0 (0.0)	0 (0.0)	0 (0.0)	2 (0.2)	0 (0.0)
Allergic reaction (%)	1 (0.3)	2 (3.4)	1 (0.1)	1 (1.1)	0 (0.0)	0 (0.0)	0 (0.0)	0 (0.0)
Wheezing (%)	2 (0.6)	0 (0.0)	0 (0.0)	2 (2.1)	1 (0.3)	0 (0.0)	0 (0.0)	0 (0.0)
Myocarditis (%)	3 (0.9)	0 (0.0)	0 (0.0)	0 (0.0)	0 (0.0)	0 (0.0)	0 (0.0)	0 (0.0)
Swelling of face, lips or throat (%)	1 (0.3)	0 (0.0)	2 (0.2)	0 (0.0)	0 (0.0)	0 (0.0)	0 (0.0)	0 (0.0)
Worsening/new diabetic symptoms (%)	0 (0.0)	0 (0.0)	2 (0.2)	0 (0.0)	0 (0.0)	0 (0.0)	2 (0.2)	0 (0.0)
Bell's Palsy (%)	0 (0.0)	0 (0.0)	1 (0.1)	0 (0.0)	0 (0.0)	0 (0.0)	0 (0.0)	0 (0.0)
Blood clot (%)	0 (0.0)	1 (1.7)	0 (0.0)	0 (0.0)	0 (0.0)	0 (0.0)	1 (0.1)	0 (0.0)
Blood clot in brain (%)	0 (0.0)	0 (0.0)	0 (0.0)	0 (0.0)	0 (0.0)	0 (0.0)	0 (0.0)	0 (0.0)
Other (%)	6 (1.7)	1 (1.7)	15 (1.1)	3 (3.2)	4 (1.4)	1 (2.5)	20 (1.6)	1 (1.5)
Don't Know (%)	5 (1.4)	0 (0.0)	8 (0.6)	2 (2.1)	6 (2.1)	0 (0.0)	10 (0.8)	3 (4.4)

Table S3. Description of SARS-CoV-2 breakthrough infections observed among vaccinated people with Down syndrome.

Infections between 1st and 2nd dose (N=13)	Infections after 2nd dose (N=8)
• Age range between 20 and 42 years<br>• Time between dose and infection<br>○ 4 within 14 days<br>○ 2 within 15–30 days<br>○ 1 within 31–45 days<br>○ 1 within 46–60 days<br>○ 5 more than 60 days<br>• Manufactures<br>○ 3 BNT162b2<br>○ 7 ChAdOx1 nCoV-19<br>○ 1 Sinovac<br>○ 2 Unknown<br>• Treatment<br>○ 2 were hospitalized<br>▪ 23 days in hospital<br>• 10 in ICU<br>▪ 18 days in hospital<br>• Outcome<br>○ 3 still had symptoms at time of survey (out of hospital)<br>○ 2 had no symptoms but tested positive<br>○ 8 recovered	• Age range between 16 and 47<br>• Time between dose and infection<br>○ 7 more than 60 days<br>○ 1 unknown<br>• Manufactures<br>○ First dose<br>▪ All 8 BNT162b2<br>○ Second dose<br>▪ 7 BNT162b2<br>▪ 1 Unknown<br>• None hospitalized<br>• Outcome<br>○ 2 still had symptoms at time of survey<br>○ 1 has had no symptoms but tested positive<br>○ 5 have recovered

Table S4 Factors associated with being unvaccinated by choice.

	N	OR	95% CI	P-VALUE
Age (per 5 years)[1]	2026	0.70	(0.61, 0.82)	<0.001
Male[2]	2026	1.01	(0.68, 1.51)	0.964
Country/region of residence[3]	2026			
USA (reference)		–	–	–
Europe[4]		0.59	(0.36, 0.95)	0.033
Canada		0.96	(0.45, 1.87)	0.912
Brazil		0.00	(0.00, NA[8])	NA[8]
Other[5]		1.28	(0.64, 2.40)	0.459
Residential setting[6]	2026	0.55	(0.16, 1.44)	0.277
Level of intellectual disabliity[7]	1940			
Borderline/ mild (reference)		–	–	–
Moderate		0.75	(0.47, 1.24)	0.249
Severe/profound		0.82	(0.41, 1.60)	0.568
Prior SARS-CoV-2 infection[7]	2018	3.30	(1.91, 5.55)	<0.001
Number of comorbidities[7]	1451	1.02	(0.83, 1.23)	0.850
Obesity[7]	1810	0.91	(0.53, 1.51)	0.717
Obstructive sleep apnea[7]	1770	0.61	(0.35, 1.03)	0.075
Gastroesophageal reflux[7]	1788	0.96	(0.66, 1.59)	0.916
Influenza vaccination[7]	1702	0.08	(0.05, 0.14)	<0.001
Pneumococcal vaccination[7]	1345	0.42	(0.24, 0.70)	0.001
HPV vaccination[7]	1328	0.24	(0.12, 0.43)	<0.001

Abbreviations: OR: odds ratio; 95% CI: 95% confidence interval

1: Adjusted for country, gender, residence

2: Adjusted for age, country, residence

3: Adjusted for age, gender, residence

4: See Table 1 for details on how many participants were included from which European country

5: Australia (N=25), Colombia (N=4), India (N=104), Mexico (N=6), Saudi Arabia (N=5) and other countries with less than 4 participants.

6: Adjusted for age, gender, country. Living at home with family (reference group) versus other living conditions (see Table 1 for details on the other categories).

7: Adjusted for age, gender, country, residence

Comorbid sum was the total number of comorbidities (Obesity, Alzheimer Thyroid disorder, Epilepsy, Blood cancer, Other cancers, Immuno-compromised, Obstructive sleep apnea, Hypertension, Diabetes, Cerebrovascular disease, Coronary heart disease, Chronic renal disease, Chronic liver disease, Chronic lung disease, Celiac, Gastroesophageal reflux, irritable bowel syndrome, and Hepatitis B)

8: Brazil had zero responses of being unvaccinated by choice
